# Thermally Stable Magneto-Plasmonic Nanoparticles for SERS with Tunable Plasmon Resonance

**DOI:** 10.3390/nano12162860

**Published:** 2022-08-19

**Authors:** Lina Mikoliunaite, Martynas Talaikis, Aleksandra Michalowska, Jorunas Dobilas, Voitech Stankevic, Andrzej Kudelski, Gediminas Niaura

**Affiliations:** 1 Department of Organic Chemistry, Center for Physical Sciences and Technology (FTMC), Sauletekio Av. 3, LT-10257 Vilnius, Lithuania; 2 Department of Physical Chemistry, Faculty of Chemistry and Geosciences, Vilnius University, Naugarduko St. 24, LT-03225 Vilnius, Lithuania; 3 Faculty of Chemistry, University of Warsaw, Pasteura St. 1, 02-093 Warsaw, Poland; 4 Department of Functional Materials and Electronics, Center for Physical Sciences and Technology (FTMC), Sauletekio al. 3, LT-10257 Vilnius, Lithuania

**Keywords:** SERS, magneto-plasmonic nanostructures, magnetite, silver nanoparticles

## Abstract

Bifunctional magneto-plasmonic nanoparticles that exhibit synergistically magnetic and plasmonic properties are advanced substrates for surface-enhanced Raman spectroscopy (SERS) because of their excellent controllability and improved detection potentiality. In this study, composite magneto-plasmonic nanoparticles (Fe_3_O_4_@AgNPs) were formed by mixing colloid solutions of 50 nm-sized magnetite nanoparticles with 13 nm-sized silver nanoparticles. After drying of the layer of composite Fe_3_O_4_@AgNPs under a strong magnetic field, they outperformed the conventional silver nanoparticles during SERS measurements in terms of signal intensity, spot-to-spot, and sample-to-sample reproducibility. The SERS enhancement factor of Fe_3_O_4_@AgNP-adsorbed 4-mercaptobenzoic acid (4-MBA) was estimated to be 3.1 × 10^7^ for a 633 nm excitation. In addition, we show that simply by changing the initial volumes of the colloid solutions, it is possible to control the average density of the silver nanoparticles, which are attached to a single magnetite nanoparticle. UV-Vis and SERS data revealed a possibility to tune the plasmonic resonance frequency of Fe_3_O_4_@AgNPs. In this research, the plasmon resonance maximum varied from 470 to 800 nm, suggesting the possibility to choose the most suitable nanoparticle composition for the particular SERS experiment design. We emphasize the increased thermal stability of composite nanoparticles under 532 and 442 nm laser light irradiation compared to that of bare Fe_3_O_4_ nanoparticles. The Fe_3_O_4_@AgNPs were further characterized by XRD, TEM, and magnetization measurements.

## 1. Introduction

Raman scattering is a very powerful vibrational spectroscopy tool for chemical analysis in various scientific fields [1]. However, it is also an extremely inefficient process as generally only one in 10^7^ photons will result in a Raman photon. To increase Raman scattering efficiency, modifications and improvements have been suggested, and new phenomena have been discovered. One of the most widely known is surface-enhanced Raman spectroscopy (SERS) [2]. The effect was discovered almost 50 years ago [3,4,5] and subsequently became a powerful ultra-sensitive vibrational spectroscopy tool with a wide range of applications. The main mechanism responsible for the amplification of the Raman signal of molecules adsorbed or located nearby the surface is electromagnetic enhancement, originating due to the generation of local surface plasmon resonance by various shapes and sizes of metal nanostructures (usually silver and gold). The increase in the efficiency of the generation of the Raman signal in the SERS effect is roughly proportional to a fourth power of the enhancement of the electric field [6]. The example theoretical simulations of the field, enhanced for plasmonic systems and the more complex systems composed from plasmonic and non-metallic parts, can be found in the following contributions: [6,7,8,9,10] and [11,12,13,14,15], respectively. In addition, a chemical enhancement mechanism operates in many cases. The obtained enhancement could be achieved up to 10^8^ times [2]. This method was suggested for use in the trace-level detection of explosives [16], the discrimination between bacteria and bacteriophages [17], cancer detection [18], and many other fields [2,19,20,21].

However, the progress of employing SERS techniques in real-world analytical and bioanalytical applications is hindered by the difficulty in simultaneously ensuring high sensitivity, efficiency, repeatability, and reproducibility [22,23]. The key role in SERS is played by the substrate responsible for providing the electromagnetic enhancement, which depends on the nanomaterial’s nature, size, shape, and structure. Recently, an interesting approach was suggested for the improvement of the SERS performance, involving the incorporation of magnetic properties to the SERS substrate system [24,25,26,27]. One of the main SERS challenges is related to measuring an especially low concentration of the samples, reaching 10^−9^ M or even a single molecule detection. In these measurements, the problem is usually to have at least some analyte molecules under the laser light, and the magnetic properties of the SERS substrate could help with this process. If the plasmonic structures have additional magnetic properties, the external magnetic field could be used for the concentration of the sample and lead particles, together with the analyte, in a specific place where the Raman signal could be registered [28,29]. There is another issue that magnetic properties are reported to solve—the coffee ring effect, obtained during the sample drying. When using the outer magnetic field during the drying process, a more uniform substrate could be obtained [30]. For these purposes, magnetic nanoparticles covered with a silver or gold layer were suggested. Importantly, the functional properties of these hybrid nanomaterials depend considerably on the form, composition, and structure of the nanoparticles [24,25,26,31,32,33,34]. The outer layer could be solid or made of smaller nanoparticles and also have a SiO_2_ interlayer or isolation shell on top. These magneto-plasmonic nanoparticles could be obtained by a few different techniques. Firstly, the magnetic core is synthesized. The most popular and easily obtained material is magnetite (Fe_3_O_4_) [27,28,29,30,31,32,33,34,35,36,37,38,39,40,41,42,43,44]. However, other materials could also be used: Fe [45,46], Co [47], Ni [48], and others [49]. Magnetite is obtained using some iron salts and other compounds, for example: NaOH [38,50,51], NaBH_4_ [45], ammonia [36,41,52] in water or solvothermal [37,40], sonochemial synthesis [35], and other methods [46,53,54,55] could be used. The plasmonic layer could be solid [38,45,50,51,53] or composed of nanoparticles [35,40,46,49,52,54], which later can be grown to a shell [37,55] or spikes [56]. Moreover, the intermediate layer of SiO_2_ could be used [35,37,41,46,52,55], as silver and gold do not easily adsorb on the surface of magnetite. The solid layer of silver or gold is usually obtained by the reduction of silver and gold salts [36,37,57] or by adsorbed seed growth [38,51]. If a layer is composed of plasmonic nanoparticles, then they are synthesized separately and later attached to the surface of the magnetite. In order to have a more stable system, an outer SiO_2_ layer could also be used [58]. These composited nanoparticles were also suggested for use in other purposes, such as the antimicrobial/antibacterial [37,49,52] or catalytical [35,40] removal or degradation of some materials [36,50,59,60]. What is more, an inverted system, where a gold nanoparticle is covered with magnetite, is also suggested [61] for Raman analysis.

We report on the synthesis and SERS performance of magneto-plasmonic nanoparticles with tunable SERS enhancement. These nanoparticles solve several problems: they have magnetic properties that help to concentrate the sample and avoid the “coffee-ring” effect while drying, as well as provide a unique possibility to tune the absorption maximum of the plasmonic band. It means one can easily form homogeneous layers from this magneto-plasmonic composite using a very simple procedure: the deposition of a layer of a sol of such a composite and the evaporation of the solvent after placing the sample in a strong magnetic field. The advantage of this method of obtaining homogeneous films for SERS measurements compared to the conventional method is a very low cost of substrate production—there is no need to use any complicated devices, such as a device for metal vapor deposition. The obtained composite was characterized with transmission electron microscopy, UV-Vis spectroscopy, XRD analysis, magnetization measurements, and Raman microscopy. The higher thermal stability of the decorated nanoparticles in comparison to the bare magnetite was determined in the presence of 532 and 442 nm laser radiation. The attached silver nanoparticles prevent the magnetite from overheating induced crystal structure change. In addition to this, the amount of AgNPs adsorbed on the surface of the magnetite could be controlled by the initial silver NP concentration. This also affects the plasmonic band position of the composite. The more silver nanoparticles are adsorbed on the surface of the magnetite, the more the red shift of the absorption maxima is noticed. In our experiment, the obtained interval was from 470 nm to more than 800 nm. This ability to adjust the absorption wavelength maximum is severely important in the SERS measurements where only one excitation wavelength is available or the sample has an absorption in the visible or near IR spectral range.

## 2. Materials and Methods

### 2.1. Materials

For the synthesis of the nanoparticles, iron sulphate heptahydrate (FeSO_4_·7H_2_O), sodium borhydride (NaBH_4_), and polyethylenimine (PEI, branched *M*_W_ 25,000) were obtained from Aldrich Chemistry (St. Louis, MO, USA); silver nitrate (AgNO_3_) was from Fluka/ Honeywell (Seelze, Germany), while sodium hydroxide (NaOH), potassium nitrate (KNO_3_), and sodium citrate (C_6_H_5_Na_3_O_7_·2H_2_O (NaCit)) were purchased from POCH S.A. (Gliwice, Poland). 4-Mercaptobenzoic acid (4-MBA; 99%) was purchased from Sigma-Aldrich (St. Louis, MO, USA). In all the experiments, ultra-pure water (resistivity 18 MΩ cm) was used.

### 2.2. Synthesis of Magnetic Nanoparticles (Fe_3_O_4_)

The synthesis of the magnetic nanoparticles was adopted from [62]. Briefly, 0.175 g of FeSO_4_·7H_2_O was dissolved in 20 mL of distilled water, purged with N_2_ gases. Later, 2.5 mL of 2 M KNO_3_ and 2.5 mL of 1 M NaOH were added to the solution, followed by 5 mL of 8 mg/mL PEI solution. The prepared mixture was heated to 90 °C and kept for 2 h. N_2_ flow and magnetic stirring were used all the time.

### 2.3. Synthesis of Silver Nanoparticles (AgNPs)

The silver nanoparticles were synthesized using the method suggested by S. Wojtysiak and A. Kudelski [63]. In brief, a 100 mL solution containing 9.1 × 10^−4^ M AgNO_3_ and 2.81 × 10^−3^ M NaCit was placed in a 250 mL two-neck round bottom flask. Nitrogen was purged for 20 min, and continuous magnetic stirring was applied. After this, 20 times 10 μL of 0.2 M NaBH_4_ solution was added to the flask, and the solution immediately turned dark yellow. Nitrogen flow and stirring were kept up for another 15 min. Later, the solution was kept in the fridge for further use.

### 2.4. Decoration of Fe_3_O_4_ with AgNPs

The decoration of the magnetic nanoparticles with AgNPs was achieved by mixing a certain amount of as-prepared AgNPs with distilled water to a final volume of 5 mL, and to this, 400 μL of Fe_3_O_4_ sol was added. All the prepared solutions are listed in Table 1. The solutions were placed in an ultrasound and kept there for 45 min. The decorated nanoparticles (Fe_3_O_4_@AgNPs) were collected with a strong magnet and washed with distilled water 3 times. In the table, the silver nanoparticle ratio to the magnetite NPs was also calculated and displayed in the last column.

### 2.5. Characterization

The characterization of the decorated NPs was performed with TEM (Talos F200X electron microscope working at an accelerating voltage of 200 kV, Thermo Fisher, Waltham, MA, USA), UV-Vis spectroscopy (spectrometer Cary 5000, Agilent, Santa Clara, CA, USA), and a benchtop powder X-ray diffraction spectrometer (MiniFlex, Rigaku, Tokyo, Japan). The synthesized silver NPs were also visualized using SEM (SU70, Hitachi, Tokyo, Japan). Thermal stability during the Raman measurements was tested using SNOM apparatus (Alpha300R, WiTec, Ulm, Germany), equipped with a 532 nm laser source, an 1800 lines/mm grating, and a 20×/0.4 NA objective. Different laser power was used, and for this reason, the signal acquisition time was also adjusted. For 10 and 83 μW, the signal acquisition time was 250 s; for 500 and 1500 μW—125 s was chosen; and for 4800 μW—25 s was chosen. All the spectra were normalized to 1 in order to better show the magnetite transition process.

For the multiwavelength Raman characterization of the nanoparticles, an inVia Raman spectrometer (Renishaw, Wotton-under Edge, UK) equipped with a confocal Leica microscope and a thermoelectrically cooled (−70 °C) CCD camera was used. The excitation sources were 442 nm (with 2400 lines/mm grating and laser power of 220 μW), 532 nm (1800 lines/mm; 45 μW), 633 nm (1800 lines/mm; 47 μW), 785 nm (1200 lines/mm; 44 μW), and 830 nm (830 lines/mm; 76 μW). Laser radiation was focused on a sample using a 50×/0.75 NA objective lens; the acquisition time was 3 s, and the final spectra were obtained by averaging 50–60 spectra from different locations on the sample. In addition, 1064 nm excitation was realized by using the FT-Raman spectrometer MultiRAM (Bruker Optik, Ettlingen, Germany), equipped with a liquid nitrogen cooled Ge diode detector. The power of the Nd:YAG laser was restricted to 10–50 mW and focused to a 100 μm in diameter spot on the sample. The spectral resolution was 4 cm^−1^, and the aperture was 4 mm; final spectra were obtained by averaging 5 spectra from different locations on a surface with 100 interferogram scans each. In SERS experiments, 2 mM aqueous 4-MBA solution was used to adsorb nanoparticles with analyte. To calculate the SERS enhancement profiles, the SERS spectral intensity was divided by the Raman spectral intensity of a 4-MBA (0.1 M) ethanol solution, I_SERS_/I_Raman_. The Raman intensities of 4-MBA were scaled down so that the laser powers and acquisition times would match those in the SERS setup; the obtained ratios were normalized to 1. We assumed that the ratio of molecules probed during the SERS and Raman measurements were wavelength independent, N_Raman_/N_SERS_ = constant.

## 3. Results and Discussion

### 3.1. Electronic Absorption Measurements

**Figure 1:**
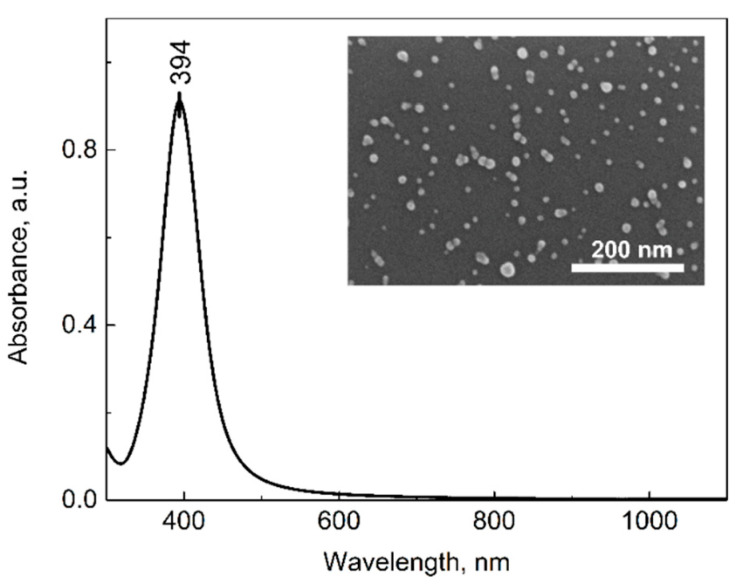
Absorption spectrum of synthesized silver NPs. SEM image of the nanoparticles on Si substrate is presented in the inset. Scale bar represents 200 nm.

The separately synthesized silver nanoparticles were first characterized and later used for magnetite decoration. From many existing synthesis routes, a few were tested, and the procedure with the most stable and the smallest resulting nanoparticles was chosen. The synthesis procedure is described in the Materials and Methods section, and the obtained nanoparticles were stable in the fridge for at least 20 days. The AgNPs were characterized using SEM and UV-Vis absorption techniques (Figure 1). As can be seen in Figure 1, the spectral maximum of the bare silver nanoparticles is at 394 nm. This, according to the literature, corresponds to nanoparticles sized between 10 nm and 20 nm [64]. The SEM image of the silver nanoparticles adsorbed on the Si substrate is shown in the inset of Figure 1. The nanoparticles are quite homogeneous; the calculated size with the ImageJ program is 13 ± 3 nm. These results are in good agreement with the literature data.

**Figure 2:**
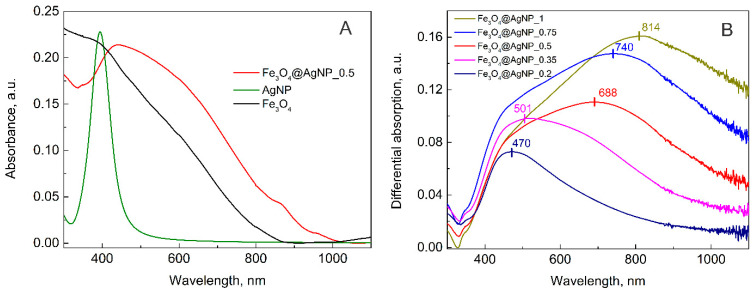
(**A**) Absorption spectrum of magnetite NPs (black), silver NPs (green), and magnetite NPs decorated with silver NPs (Fe_3_O_4_@AgNP_0.5; red); (**B**) the differential spectra of various amounts of AgNP-decorated magnetite, calculated by subtracting magnetite absorption spectra from each sample with decorated NPs.

The characterized nanoparticles were later used for the decoration of the magnetic NPs. According to the literature and our observation, the synthesized magnetite nanoparticles were around 50 nm in diameter. As the idea was to decorate it with plasmonic nanoparticles, their size should be remarkably smaller in comparison to the magnetite. It is known that the increase in the size of the plasmonic nanoparticle will shift the absorption peak to the longer wavelength. This will also affect the SERS enhancement factor. Other authors suggest that increasing the particle size produces a stronger LSPR signal [65]. However, the idea of this manuscript was to decorate the magnetite crystal with plasmonic nanoparticles. As the size of magnetite is 50 nm, the silver nanoparticles should be smaller (for example 13 nm); in this way, more hotspots could be achieved between the silver nanoparticles. This has already been shown on polystyrene spheres. Smaller distances between the nanoparticles result in a higher SERS signal [66]. Thus, 13 nm-sized citrate-stabilized AgNPs were expected to work well. The UV-Vis spectra of magnetite, the bare AgNPs, and the magnetite decorated with silver NPs are presented in Figure 2A. For magnetite, a gradual increase of the spectrum towards a shorter wavelength is observed, while for decorated NPs a broad plasmonic peak is also observed.

The decoration of magnetite with AgNPs was performed using different amounts of AgNP solution. As described in the Materials and Method section, the ratio of AgNPs to Fe_3_O_4_ was varied by diluting the Ag colloid solution with ultrapure H_2_O from an initial as-synthesized concentration (100%) to 20% and then adding 0.4 mL of a fixed concentration of Fe_3_O_4_. For all these samples, the UV-Vis spectra were measured and presented in the supporting information (Figure S1). All the colloid solutions exhibited a typical scattering background that exponentially increased towards shorter wavelengths; therefore, to minimize the background contribution and to accentuate the plasmonic profiles of the nanoparticles, differential spectra were calculated by subtracting the magnetite absorption spectrum from the composite nanoparticle absorption spectra. The obtained result is presented in Figure 2B. As can be seen, due to the increase of the AgNP amount the absorption spectra is changing. At the lowest amount of silver NPs, the most intensive plasmonic peak is observed at around 470 nm, with a slope to longer wavelengths, indicating an additional low-intensity peak. With the increasing of the AgNP sol volume in the “Ag decoration solution” from 35% to 50%, 75%, and 100%, the absorption peak red-shifts from 501 to 688, 740, and 814 nm, respectively. The shift is related to the intensification of the spectral component at longer wavelengths. When the amount of AgNPs in the final solution is more than 50% by volume, the second peak outgrows the one at the shorter wavelengths. The first peak at 470 nm could be attributed to the silver NP plasmons. In solution, this peak was at 394 nm; however, after adsorption on the magnetite the peak broadens and shifts to the longer wavelength. This was also noticed by other scientists and attributed to the agglomeration of plasmonic nanoparticles [58,67]. When the number of AgNPs on the surface of Fe_3_O_4_ is increasing they start to interact with each other, and due to that, an additional plasmonic peak appears. In addition to this, a red shift of the latter peak is seen. This effect was described in the publication of Ma et al. [68], where they theoretically calculated the local surface plasmon resonance properties of two or three silver nanospheres at different gap distances. It was concluded that the closer the two spheres are to each other, the more the red-shifted second plasmonic peak is obtained [68]. In this case, one could change the plasmonic maxima of the decorated nanoparticles simply by changing the amount of AgNPs used for the decoration procedure.

### 3.2. XRD Analysis

**Figure 3:**
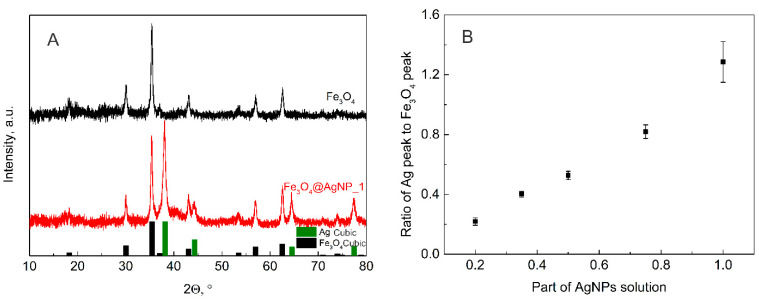
(**A**) XRD diffractograms of magnetite NPs (black) and magnetite NPs decorated with the highest amount of AgNPs (Fe_3_O_4_@AgNP_1; red); Crystallography Open Database files of magnetite (No. 9007644) and silver (No. 5000218) are presented in black and green columns, respectively. (**B**) Ratio of the main magnetite (35.4°) and silver (38.2°) peak intensities calculated for the different volume of AgNP solution used in decoration of magnetite.

XRD analysis was also performed on all the decorated magnetite samples with the expectation of seeing the dependence of the silver peak on the amount of solution used for the decoration procedure. All the obtained XRD results are presented in the supporting information (Figure S2); however, here the diffractograms of pure magnetite and magnetite decorated with the highest amount of silver nanoparticles are presented together with the Crystallography Open Database files of magnetite (No. 9007644) and silver (No. 5000218) (Figure 3A). The peaks of silver and magnetite in the diffractograms are visible; thus, it is possible to calculate the dependence of their intensity ratio on the amount of AgNP solution used in the decoration procedure. Two peaks were chosen for comparison, 35.4° for magnetite and 38.2° for silver. The obtained results are presented in Figure 3B. The dependence of the XRD peaks ratio on the AgNP volume is evidence of the linear relationship between the AgNP dilution with ultrapure water and the final Ag to Fe_3_O_4_ molar ratio.

### 3.3. Magnetite Stability under Laser Radiation

**Figure 4:**
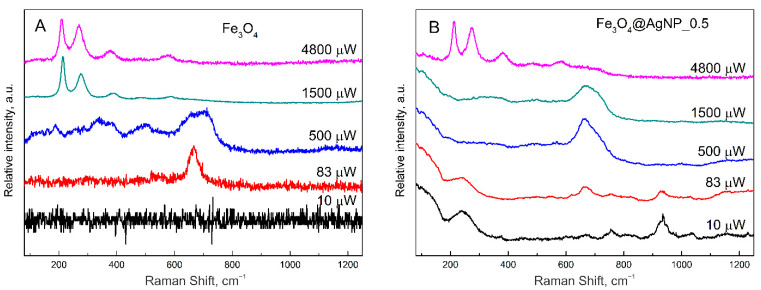
Raman spectra of (**A**) magnetite nanoparticles and (**B**) magnetite nanoparticles decorated with silver nanoparticles (Fe_3_O_4_@AgNP_0.5) at different laser powers from 10 μW to 4.8 mW. Spectra were taken at 532 nm laser light on the nanoparticle samples dried on glass substrate.

During the Raman measurements, it was noticed that the magnetic nanoparticles are more stable under laser light if silver NPs are attached. For this reason, two samples were tested at different laser powers from 10 μW to 4800 μW. The obtained results are shown in Figure 4.

In Figure 4A, the Raman spectra of bare magnetite nanoparticles are presented. As can be seen at the lowest power, no Raman signal is registered at all; at 83 μW, a characteristic Fe_3_O_4_ peak at 667 cm^−1^ is seen; however, at 500 μW a change to the maghemite is already visible by the growth of three wide peaks. Magnetite and maghemite have almost identical lattice parameters, and in the Raman spectra, the A_1g_ vibrational modes of those two materials are close to each other: 667 cm^−1^ for magnetite and 710 cm^−1^ for maghemite [69]. Thus, the change of the 667 cm^−1^ peak is related to the change of the crystallographic structure of iron oxide. Due to the change of the crystal structure, the magnetic properties are also changing. Magnetite is known to be ferromagnetic, maghemite—ferrimagnetic, and hematite weakly ferromagnetic or antiferromagnetic. Finally, at 1500 and 4800 μW, a clear hematite spectrum was registered. This heat-induced change was also observed by other scientists [70,71]. However, if the magnetite is decorated with silver nanoparticles (for this experiment the middle coverage Fe_3_O_4_@AgNP_0.5 was chosen), the crystal structure is more photo- and thermally resistant. At a laser power of 10 μW, a characteristic spectrum of silver nanoparticles enhancing a random contamination is seen. These peaks are also present at 83 μW; however, the characteristic magnetite peak at 667 cm^−1^ is also present. At higher powers (500 and 1500 μW), this characteristic peak is even more intensive, probably due to the SERS effect from the silver nanoparticles. Additionally, only at the highest power (4800 μW) the characteristic peak of hematite is present, indicating the change of magnetite crystal structure. Thus, resistivity to laser radiation at 532 nm considerably increases for hybrid magneto-plasmonic nanoparticles compared with bare Fe_3_O_4_. Due to the increased magnetite light absorbance towards the shorter wavelengths according to Figure 2A, the laser power dependency was reworked for the 442 nm excitation as well, and similarly to 532 nm, the higher photo- and thermal resistivity was recognized for composite magnetite–silver nanoparticles (Figure S3).

This advantage of increased nanoparticle stability can be explored in various spectroscopic studies. The observed findings can be associated with the thermal conductivity of the used materials. For magnetite nanoparticles, the thermal conductivity is 0.144 W/m K [72]. The bulk silver has a thermal conductivity of 406 W/m K; however, the theoretical thermal conductivity of silver nanoparticles is considerably lower, 0.425 W/m K at 300 K [73]. Thus, the difference between the silver and the magnetite nanomaterial thermal conductivity values is almost three times. Then the laser light is enlightened, the heating process starts, but in the presence of silver more heat is transferred to the surroundings from the magnetite surface and the degradation processes are slowed down.

### 3.4. Magnetic Properties of Decorated NPs

**Figure 5:**
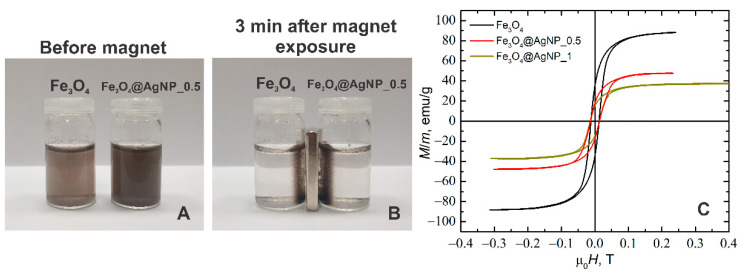
Solutions of magnetite and magnetite decorated with silver NPs (**A**) just prepared and (**B**) in 3 min after exposure to a permanent magnet; (**C**) Hysteresis loop of the magnetite (black), and magnetite decorated with silver nanoparticles: Fe_3_O_4_@AgNP_0.5 (red) and Fe_3_O_4_@AgNP_1 (dark yellow).

A study of the magnetic properties of the particles has shown that Fe_3_O_4_ particles decorated by AgNPs remain ferromagnetic. This is evidenced by the experiments using permanent magnets as well as the magnetization studies. Figure 5A,B shows an experiment in which Fe_3_O_4_ and Fe_3_O_4_@AgNP_0.5 nanoparticles were suspended in water (see Figure 5A) and placed in a permanent magnetic field (see Figure 5B). The permanent magnet in this case was placed to the side of the bottles with these solutions. As we can see, in both cases all the particles were attracted to the side of the bottles at the location of the magnet. After three minutes from the beginning of the experiment, the solution in both bottles became transparent. This proves that, using the given decoration method, all the AgNPs have adhered to the Fe_3_O_4_ particles and that the Fe_3_O_4_ particles do not lose their ferromagnetic properties after decoration.

In addition, magnetization studies of the synthesized nanoparticles were performed using a pulsed magnetic field with a duration of about 4 ms and a field strength up to 0.5 T (the measurement setup is presented in our previous work [69]). The results of these measurements are shown in Figure 5C. As can be seen, the shapes of the magnetization curves are very similar, and the coercive field for all the particles is the same, about 13 mT. However, the magnitude of magnetization saturation depends on the degree of particle decoration. For example, if for the undecorated Fe_3_O_4_ particles the magnetization saturation was 89 emu/g, then for the silver-decorated particles with a composition of Fe_3_O_4_@AgNP_0.5 it drops to 47 emu/g and for Fe_3_O_4_@AgNP_1 it drops to 36 emu/g. It is caused mainly because Ag is a diamagnetic material and changing the concentration of silver only leads to a reduction in the relative mass of Fe_3_O_4_ to the total mass of the decorated particles. Similar characteristics were obtained in other papers, for example when CoNPs [47] and FeNPs [49] were coated with AgNPs, as well as when FeNPs were coated with AuNPs [58,74].

### 3.5. SERS Measurements

**Figure 6:**
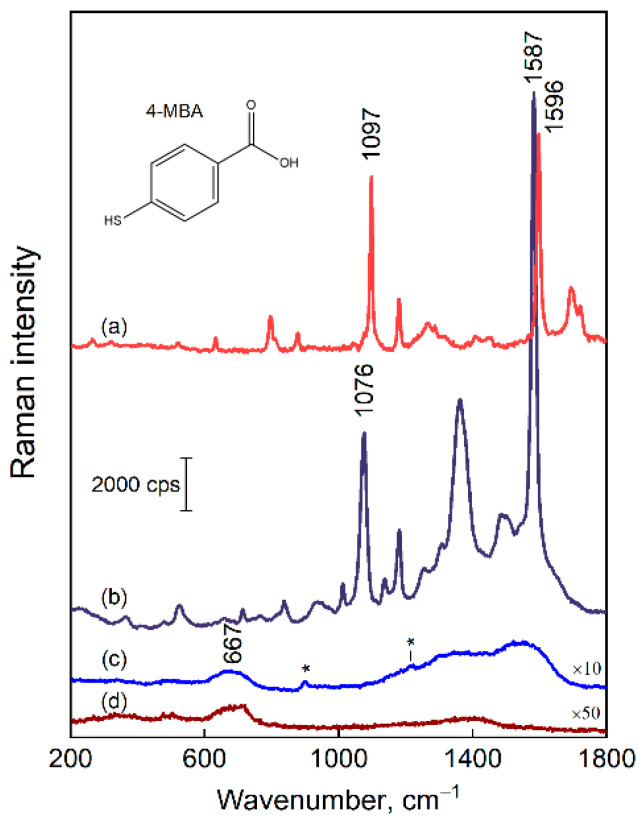
Raman-difference spectrum of 0.1 M 4-MBA in ethanol solution (ethanol spectrum is subtracted) (**a**), SERS spectrum of 4-MBA adsorbed on Fe_3_O_4_@AgNP_0.5 (**b**), SERS spectrum of bare Fe_3_O_4_@AgNP_0.5 (**c**). Notice the asterisks that mark spectral features due to contaminations. Raman spectrum of magnetite nanoparticles (**d**). The laser excitation wavelength was 532 nm. The scale bar applies for the (**b**–**d**) (notice the multipliers), whereas the intensity of (**a**) is arbitrary.

The plasmonic properties of the composite silver-decorated magnetite nanoparticles were probed by drying a colloid solution on a solid surface under a strong magnetic field, followed by adsorption of the reporter 4-mercaptobenzoic acid (4-MBA) molecule (from 2 mM in H_2_O). Figure 6 compares the 4-MBA solution (0.1 M in ethanol) spectrum with the one obtained from Fe_3_O_4_@AgNP_0.5. The SERS bands due to contaminations were of diminishing intensity when compared with the spectral features of the 4-MBA adsorbate. The spectral modes that appear due to impurities are marked with the asterisks in Figure 6c. The broad bands in the vicinity of 1360 and 1580 cm^−1^ are related to the presence of a small amount of carbon species.

**Figure 7:**
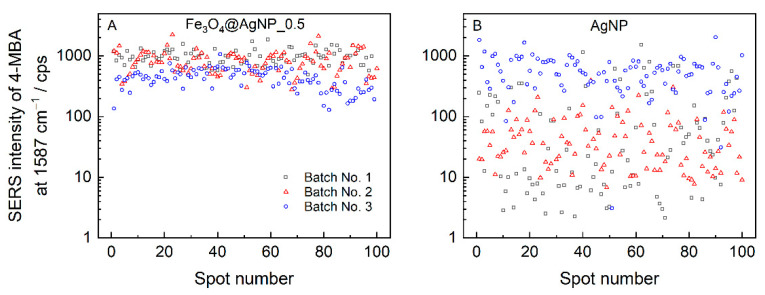
The intensity distribution of the dominant 1587 cm^−1^ band was obtained from 100 spectra of 4-MBA adsorbed on (**A**) Fe_3_O_4_@AgNP_0.5 and (**B**) AgNPs. Nanoparticles were dried while placing a glass slide in a strong magnetic field. Spectra were excited at 532 nm with 45 μW laser power focused to a spot on a surface of 1 μm in diameter and 12 s acquisition time. Three different preparations of Fe_3_O_4_@AgNP_0.5 and AgNPs are analyzed.

Drying the colloid solution droplet on a surface resulted in the accumulation of non-volatile solutes at the edge of the drop—an effect known as the “coffee ring” [75,76]. Such an accumulation leads to an uneven distribution of material, which in the case of plasmonic nanoparticles has already been shown to reduce the repeatability of the SERS signal and the overall spectral intensity [58,67]. When composite magneto-plasmonic nanoparticles are dried in a strong magnetic field, their distribution on a surface becomes highly homogeneous, as can be seen in the right-hand side image in Figure S4. Dried AgNPs and Fe_3_O_4_@AgNP_0.5 colloid droplets were adsorbed with 4-MBA and measured at 100 different locations on the sample surface; the intensities of the dominant band at 1587 cm^−1^ were plotted in Figure 7. The SERS enhancement reproducibility could be quantified by the relative standard deviation (RSD), which allows for the accessing of both the spot-to-spot reproducibility within a single sample and the sample-to-sample reproducibility amongst the different nanoparticle preparations. We found Fe_3_O_4_@AgNP_0.5 to exhibit a much greater spot-to-spot reproducibility, with values ranging from 27 to 44% for individual batches, compared to the AgNPs, with the RSD varying from 62 to 496%. Addressing the reproducibility between the different nanoparticle batches (sample-to-sample reproducibility), the intensity deviations were also higher for the bare silver nanoparticles; accordingly, the RSDs were 88% and 37% for the AgNPs and Fe_3_O_4_@AgNP_0.5.

**Figure 8:**
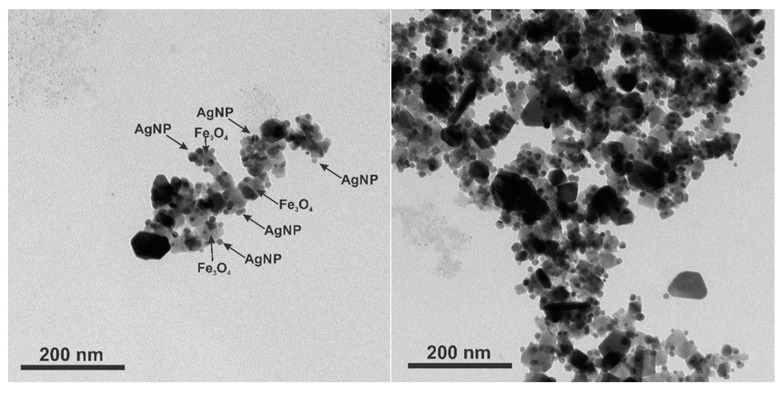
TEM images of cubic magnetite covered with spherical silver nanoparticles (Fe_3_O_4_@AgNP_0.5).

**Figure 9:**
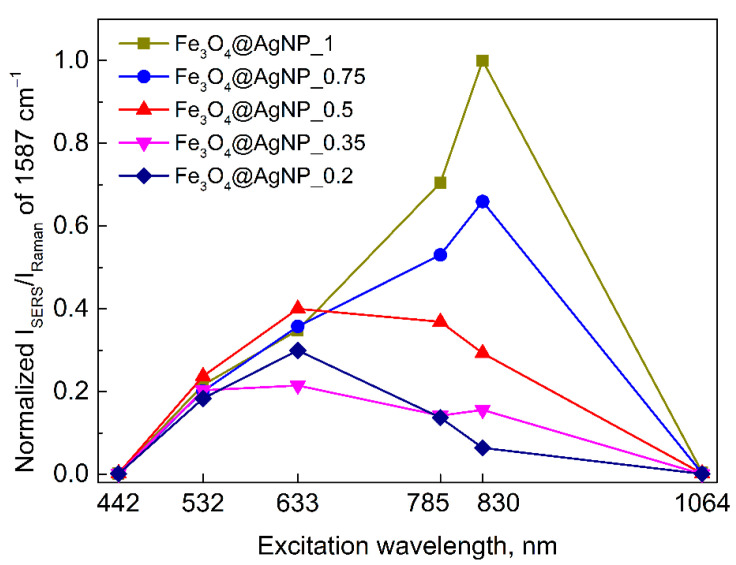
Excitation wavelength dependence of the normalized SERS intensity of 1587 cm^−1^ band from 4-MBA adsorbed on a Fe_3_O_4_@AgNP that have a varied relative concentration of AgNPs. Each data point is obtained by averaging 50–60 independent SERS measurements, except for 1064 nm excitation where 5 different measurements were averaged. The increase in available surface area for 4-MBA to adsorb and contribute to the signal with increasing relative AgNPs concentration (from sample Fe_3_O_4_@AgNP_0.2 to Fe_3_O_4_@AgNP_1) is accounted.

TEM images were made to see how the silver nanoparticles were adsorbed on the surface of the magnetite (Figure 8). The Fe_3_O_4_ nanoparticles of 50 nm average diameter are attached with 13 nm AgNPs. The ability to control the number of AgNPs per one magnetic nanoparticle by simply adding different volumes of AgNPs to the Fe_3_O_4_ solution brings a practical aspect to the ability to tune the plasmon resonance intensity and frequency to match the given laser excitation. This has already been illustrated by the optical properties in Figure 2B, and now, we present the complementing multiwavelength-SERS results. The SERS enhancement factor (*EF*) is

(1) $$EF=\frac{I_{SERS}}{I_{Raman}}\frac{N_{Raman}}{N_{SERS}}$$

where *I* and *N* are the intensity and number of probed molecules during the SERS and Raman experiments, respectively. We consider *N_Raman_*/*N_SERS_* to be excitation wavelength independent; therefore, one can compare the surface-enhancement capabilities between the different excitation wavelengths and the different compositions of Fe_3_O_4_@AgNP, just by analyzing their *I_SERS_*/*I_Raman_*. Figure 9 compares the normalized excitation profiles of 4-MBA adsorbed on a Fe_3_O_4_@AgNP that have a varied relative concentration of AgNPs. The profiles show that larger AgNP concentrations effectively increase the relative 1587 cm^−1^ band’s intensity, especially at longer wavelengths. Furthermore, a clear shift of the enhancement maxima towards the longer wavelengths can be observed. The shifting might be explained by the plasmon hybridization effect, which is highly dependent on the distance between the plasmonic materials, their shape, and the dielectric constant of the environment [77,78]. By changing the AgNP and Fe_3_O_4_ ratio in the solution, one can have control over the average distance between the plasmonic particles on a surface of larger magnetic particle, and in such a way, tune the plasmon resonance frequency.

Table 2 provides rough estimates of the enhancement factors (*EF*) calculated according to the dominant 1587 cm^−1^ band of 4-MBA for Fe_3_O_4_@AgNP_0.5 and AgNPs at laser exactions from 442 to 830 nm. The procedure to find EFs is detailed elsewhere [79].

## 4. Conclusions

In this work, magneto-plasmonic nanoparticles (Fe_3_O_4_ nanoparticles of 50 nm in diameter covered with Ag nanoparticles of 13 nm in diameter; Fe_3_O_4_@AgNP) were synthesized and studied for their possible use in surface-enhanced Raman spectroscopy. We have shown that simply by varying the initial volumes of the Fe_3_O_4_ and AgNP colloid solutions, we were able to manipulate the density of the AgNPs adsorbed on a single magnetite nanoparticle surface. The benefit of such a composition of Fe_3_O_4_@AgNPs is manifold. First, under a strong magnetic field the nanoparticles arrange in a more homogeneous way, which results in higher SERS signal enhancement and spectral reproducibility within the sample and between the batches, compared to bare silver nanoparticles. Second, the plasmon resonance frequency and intensity could be readily manipulated by simply changing the ratio between the magnetic and plasmonic parts of the composite, which allows for the tuning of the nanoparticles to a specific laser excitation wavelength in the SERS setup. Generally, the higher the AgNPs number per one Fe_3_O_4_ nanoparticle, the more red-shifted the plasmon resonance frequency and the higher its intensity. Third, these nanoparticles exhibited greater photo- and thermal stability under 532 and 442 nm light irradiation compared to the bare magnetite NPs. We estimated the SERS enhancement factors of Fe_3_O_4_@AgNP_0.5 hybrid nanoparticles for five laser excitations, with the highest found for 633 nm to be 3.1 × 10^7^. Moreover, the advantage of the method used for obtaining homogeneous films for the SERS measurements compared to the conventional methods is a very low cost of substrate production – there is no need to use any complicated devices, such as a device for metal vapor deposition. One can easily form homogeneous layers from this magneto–plasmonic composite using a very simple procedure: deposition of a layer of a sol of such a composite and evaporation of the solvent after placing the sample in a strong magnetic field.

## Figures and Tables

**Table 1:** AgNP, H_2_O, and Fe_3_O_4_ volumes (mL) used in this study to form Fe_3_O_4_@AgNPs with varied silver and magnetite ratio. The ratio of AgNP to Fe_3_O_4_ NP is calculated in the last column.

	$V_{AgNP},\;\mathrm{mL}$	$V_{H_{2}O},\;\mathrm{mL}$	$V_{Fe_{3}O_{4}},\;\mathrm{mL}$	Ratio of AgNP to Fe_3_O_4_ NP
Fe_3_O_4_@AgNP_1	5	0	0.4	16.1
Fe_3_O_4_@AgNP_0.75	3.75	1.25	0.4	12.1
Fe_3_O_4_@AgNP_0.5	2.5	2.5	0.4	8.1
Fe_3_O_4_@AgNP_0.35	1.75	3.25	0.4	5.6
Fe_3_O_4_@AgNP_0.2	1	4	0.4	3.2

**Table 2:** Estimated enhancement factors for Fe_3_O_4_@AgNP_0.5 and AgNPs.

Wavelength, nm	Enhancement Factor	
	Fe_3_O_4_@AgNP_0.5	Ag
442	5.4 × 10^4^	1.6 × 10^4^
532	5.0 × 10^6^	1.9 × 10^5^
633	3.1 × 10^7^	1.2 × 10^6^
785	2.4 × 10^6^	5.5 × 10^4^
830	4.3 × 10^5^	1.3 × 10^4^

## Data Availability

All data supporting the findings of this study are available from the corresponding author upon reasonable request.

## Supplementary Materials

The following supporting information can be downloaded at: https://www.mdpi.com/article/10.3390/nano12162860/s1, Figure S1: absorption spectra of all prepared samples: magnetite (black), magnetite decorated with various amount of silver nanoparticles: 100% (dark yellow), 75% (blue), 50% (red), 35% (magenta), and 20% (dark blue); Figure S2: XRD diffractograms of all prepared samples: magnetite (black), magnetite decorated with various amount of silver nanoparticles: 100% (dark yellow), 75% (blue), 50% (red), 35% (magenta), and 20% (dark blue); Figure S3: Raman spectra of (A) magnetite nanoparticles and (B) composite magnetite–silver nanoparticles (Fe_3_O_4_@AgNP_0.5) at varied laser powers from 0.026 mW to 2.65 mW; the 442 nm laser radiation was focused to a spot of 1 μm in diameter on the dried nanoparticle samples; Figure S4: microscope images of AgNPs (left) and Fe_3_O_4_@AgNP_0.5 (right) drops dried on a glass slide. Fe_3_O_4_@AgNP_0.5 was dried under a strong magnetic field.

## Sample Availability

Samples of the compounds are available from the authors.
