# Supplementary material for: Thermally Stable Magneto-Plasmonic Nanoparticles for SERS with Tunable Plasmon Resonance
Source: Nanomaterials (Basel). 2022 Aug 19;12(16):2860. doi: 10.3390/nano12162860 (PMC9416134; doi:10.3390/nano12162860)
Supplement: Supplementary file 1 [file nanomaterials-12-02860-s001.zip › nanomaterials-1866484-supplementary.pdf]

nanomaterials

Supplementary Materials

Thermally Stable Magneto-Plasmonic Nanoparticles for SERS with Tunable Plasmon Resonance

Lina Mikoliunaite [1,2], Martynas Talaikis [1], Aleksandra Michalowska [3], Jorunas Dobilas [4], Voitech Stankevic [4], Andrzej Kudelski [3] and Gediminas Niaura [1,*]

1 Department of Organic Chemistry, Center for Physical Sciences and Technology (FTMC), Sauletekio av. 3, LT-10257 Vilnius, Lithuania
2 Department of Physical Chemistry, Faculty of Chemistry and Geosciences, Vilnius University, Naugarduko st. 24, LT-03225 Vilnius, Lithuania
3 Faculty of Chemistry, University of Warsaw, Pasteura st. 1, 02-093 Warsaw, Poland
4 Department of Functional Materials and Electronics, Center for Physical Sciences and Technology (FTMC), Sauletekio al. 3, LT-10257 Vilnius, Lithuania
* Correspondence: gediminas.niaura@ftmc.lt

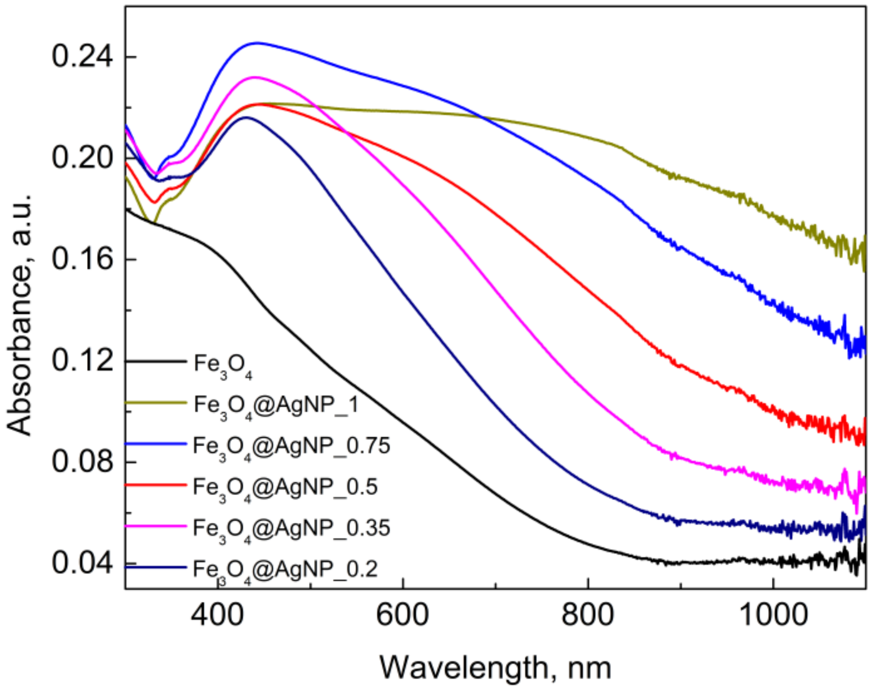

Figure S1. Absorption spectra of all prepared samples: magnetite (black), magnetite decorated with various amount of silver nanoparticles: 100% (dark yellow), 75% (blue), 50% (red), 35% (magenta), and 20% (dark blue).

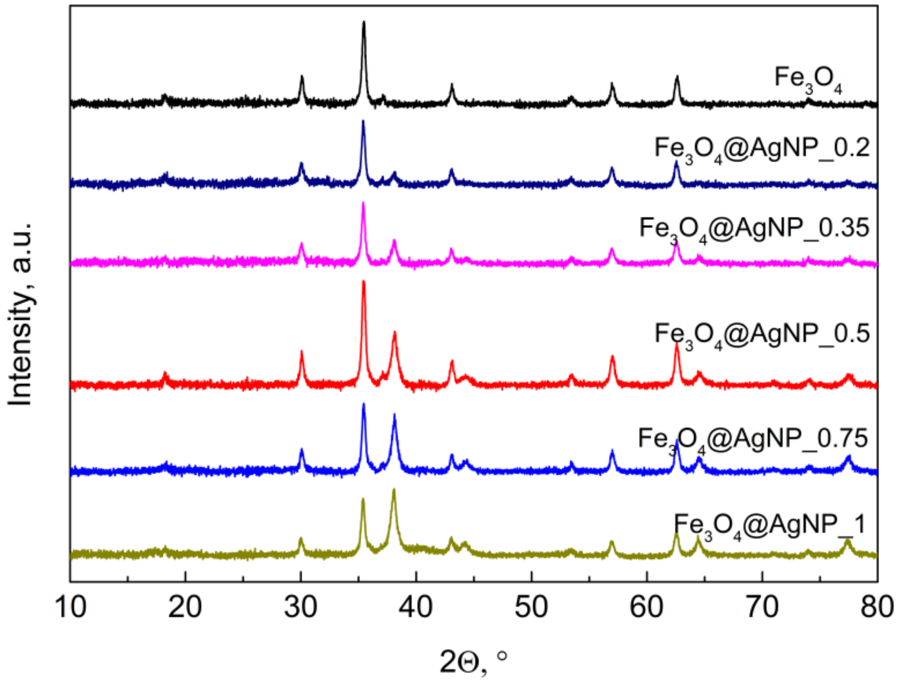

Figure S2. XRD diffractograms of all prepared samples: magnetite (black), magnetite decorated with various amount of silver nanoparticles: 100% (dark yellow), 75% (blue), 50% (red), 35% (magenta), and 20% (dark blue).

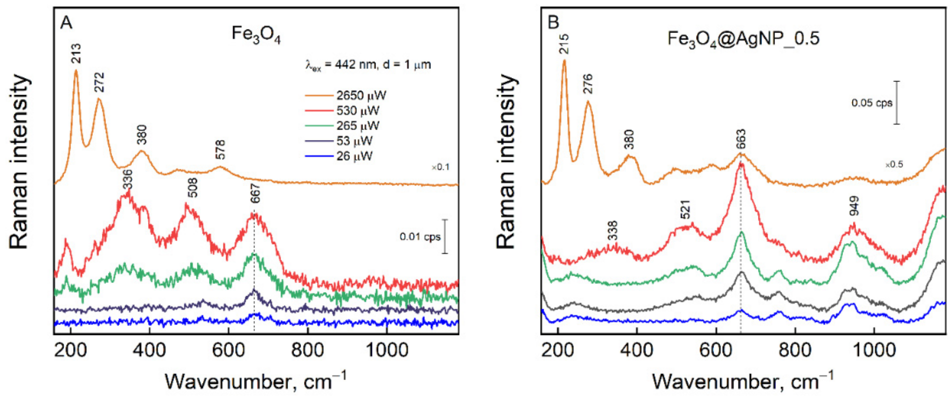

Figure S3. Raman spectra of **(A)** magnetite nanoparticles and **(B)** composite magnetite-silver nanoparticles (Fe_3O_4@AgNP_0.5) at varied laser powers from 0.026 mW to 2.65 mW. The 442 nm laser radiation was focused to a spot of 1 µm in diameter on the dried nanoparticle samples.

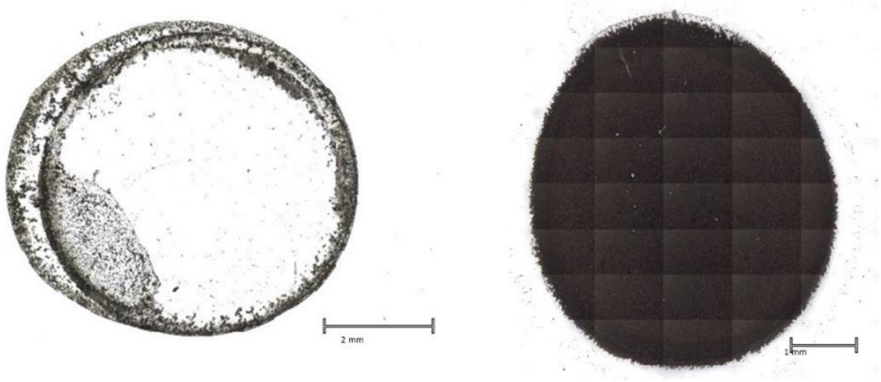

Figure S4. Microscope images of AgNPs (left) and Fe_3O_4@AgNP_0.5 (right) drops dried on a glass slide. Fe_3O_4@AgNP_0.5 was dried under strong magnetic field.
